# Supplementary material for: Identification of photoperiod-induced specific miRNAs in the adrenal glands of Sunite sheep (Ovis aries)
Source: Front Vet Sci. 2022 Jul 22;9:888207. doi: 10.3389/fvets.2022.888207 (PMC9354845; doi:10.3389/fvets.2022.888207)
Supplement: Supplementary file 1 [file Data_Sheet_1.ZIP › Supplementary file/miRDeep2 file.zip!/miRDeep2 file/Novel_Structure/SPLP42A3/Novel_227.pdf]

Provisional ID	:	NC_019471.2_24965
Score total	:	2
Score for star read(s)	:	-1.3
Score for read counts	:	0
Score for mfe	:	1.7
Score for randfold	:	1.6
Score for cons. seed	:	
Total read count	:	271
Mature read count	:	271
Loop read count	:	0
Star read count	:	0

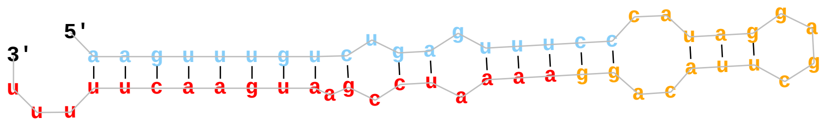

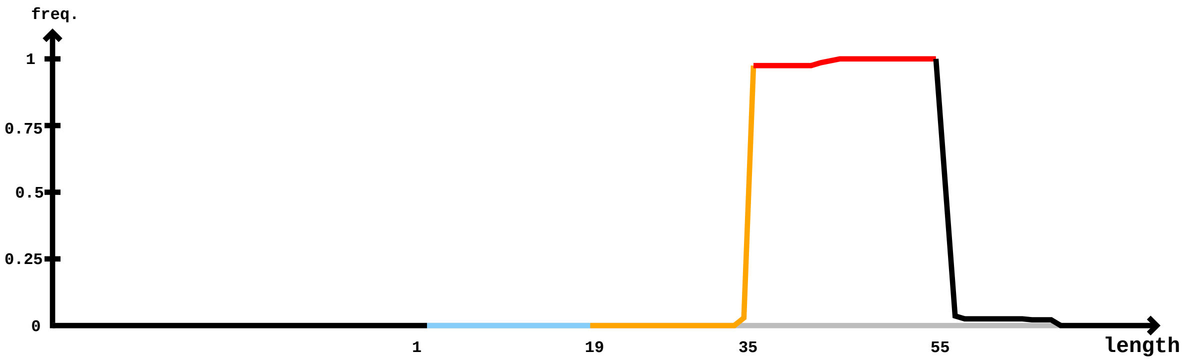

Star Mature

```
5'- cuuuuaaauuuaugcacauauguauugguuggccaaaaguuugucugaguuucccauaggagcuuacaggaaaauccgaaugaacuuuuuggccaacccauauauuggccuu   -3'    exp
    ............((.((.((((((.((((((((((((((((((((.((.(((((..(((....)))..))))).)).).)))))).)))))))))))))))))))))))...    reads      mm         sample
    .....................................................................gaaaaCccgaaugaacuuuuug.....................    6          1          seq
    .....................................................................gaaaauccgaaugaacuuuuugg....................    1          0          seq
    .....................................................................gaaaaCccgaaugaacuuuuugg....................    1          1          seq
    ......................................................................aaaauccgaaugaacuuuuC......................    1          1          seq
    ......................................................................aaaauccgaaugaacuuuuu......................    14         0          seq
    ......................................................................aaaaucUgaaugaacuuuuu......................    247        1          seq
    ......................................................................aaaauccgaaugaacuuuuugg....................    1          0          seq
    .............................................................................gaaugaacuuuuuggccaacccauU..........    3          1          seq
    ..............................................................................aaugaacuuuuuggccaaccc.............    1          0          seq
    ..............................................................................aaugaacuuuuuggccaacccaGa..........    1          1          seq
    ...............................................................................augaacuuuuuggccaacccaGa..........    1          1          seq
    ...............................................................................augaacuuuuuggccaacccaAa..........    1          1          seq
```
